# Intramedullary Spinal Cord Metastasis From Oligometastatic Melanoma of Unknown Primary Treated With Stereotactic Body Radiotherapy

**DOI:** 10.7759/cureus.116732

**Published:** 2026-09-22

**Authors:** Karen Anne Aguirre-Meneses, Justin Owen, Benjamin Mou

**Affiliations:** 1 Radiation Oncology, BC Cancer – Kelowna, Kelowna, CAN

**Keywords:** dual immunotherapy, intramedullary spinal cord metastasis, melanoma, oligometastasis, stereotactic body radiotherapy (sbrt)

## Abstract

Intramedullary spinal cord metastasis (ISCM) is an uncommon and challenging clinical entity with no established consensus regarding its optimal management. A 37-year-old female presented with left-sided facial numbness, right leg paresthesia with decreased temperature sensation, and right lower extremity weakness. Investigations demonstrated two intracranial lesions and an ISCM at T10 to T11. Biopsy of the intracranial lesion confirmed melanoma. The patient was treated with stereotactic body radiotherapy (SBRT) to a dose of 30 Gy in 5 fractions to the ISCM and stereotactic radiosurgery to a dose of 20 Gy in a single fraction to the intracranial metastases. The spinal cord dose received by a 0.035 cc volume was limited to less than or equal to 30 Gy. No spinal cord planning organ-at-risk volume was used. SBRT was followed by combination ipilimumab and nivolumab for four cycles, then maintenance nivolumab thereafter. At 25 months post-SBRT, the treated lesions decreased in size. There were no severe acute or late radiation-related toxicities, and the patient remained fully ambulatory from presentation to last follow-up.

## Introduction

Intramedullary spinal cord metastasis (ISCM) is rare and accounts for 1-3% of intramedullary spinal cord tumors [1]. The most common primary malignancies associated with ISCM are breast (41.7%), lung (18.3%), melanoma (8.3%), and renal cell carcinoma (6.7%) [2]. Melanoma of unknown primary represents approximately 3.2% of all metastatic melanoma cases and typically involves the lymph nodes, followed by subcutaneous tissue and the visceral organs [3]. Oligometastatic melanoma of unknown primary initially presenting as an ISCM is exceptionally rare, with only one published case report to date, to our knowledge [4]. ISCM presentation can precede the diagnosis of the primary tumor, typically with symptoms such as weakness, sensory symptoms, bladder or bowel obstruction, or pain [5]. At diagnosis, data regarding the co-occurrence of ISCM and intracranial metastases from melanoma remain limited.

There is no established consensus for the management of ISCM due to the rarity of the disease [6]. Most patients typically receive palliative treatment in the form of systemic therapy, radiotherapy, surgery in selected cases, or a combination of systemic therapy and local therapy, with the goal of alleviating symptoms and preserving neurologic function [6]. With prolonged survival of patients with oligometastatic melanoma treated with immunotherapy [7], stereotactic body radiotherapy (SBRT) offers potential synergistic benefits and durable local control. However, the optimal combination of prescription dose and spinal cord dose constraint, including use of a planning organ-at-risk volume (PRV), remains unclear in the setting of ISCM. Treatment planning is particularly challenging for ISCM due to the competing dosimetric goals of maximizing dose to the tumor and sparing the spinal cord when the target lies directly within the organ at risk. This case report demonstrates the feasibility of SBRT for the management of oligometastatic melanoma of unknown primary presenting with ISCM and synchronous brain metastases. This work has not been previously presented at any meeting, conference, or academic forum.

## Case presentation

A 37-year-old female presented with left-sided facial numbness, right leg paresthesia with decreased temperature sensation, and right lower extremity weakness, suggestive of incomplete spinal cord injury. Past medical history included endometriosis and a loop electrosurgical excision procedure for a benign cervical neoplasm. Her Zubrod performance status was 0. She had no family history of malignancy and no prior history of radiotherapy.

MRI of the spine demonstrated a 12 mm × 9 mm × 10 mm intensely enhancing mass within the thoracic cord at the T10 to T11 level, centered within the cord without bulging through the left anterior margin (Figure 1).

**Figure 1:**
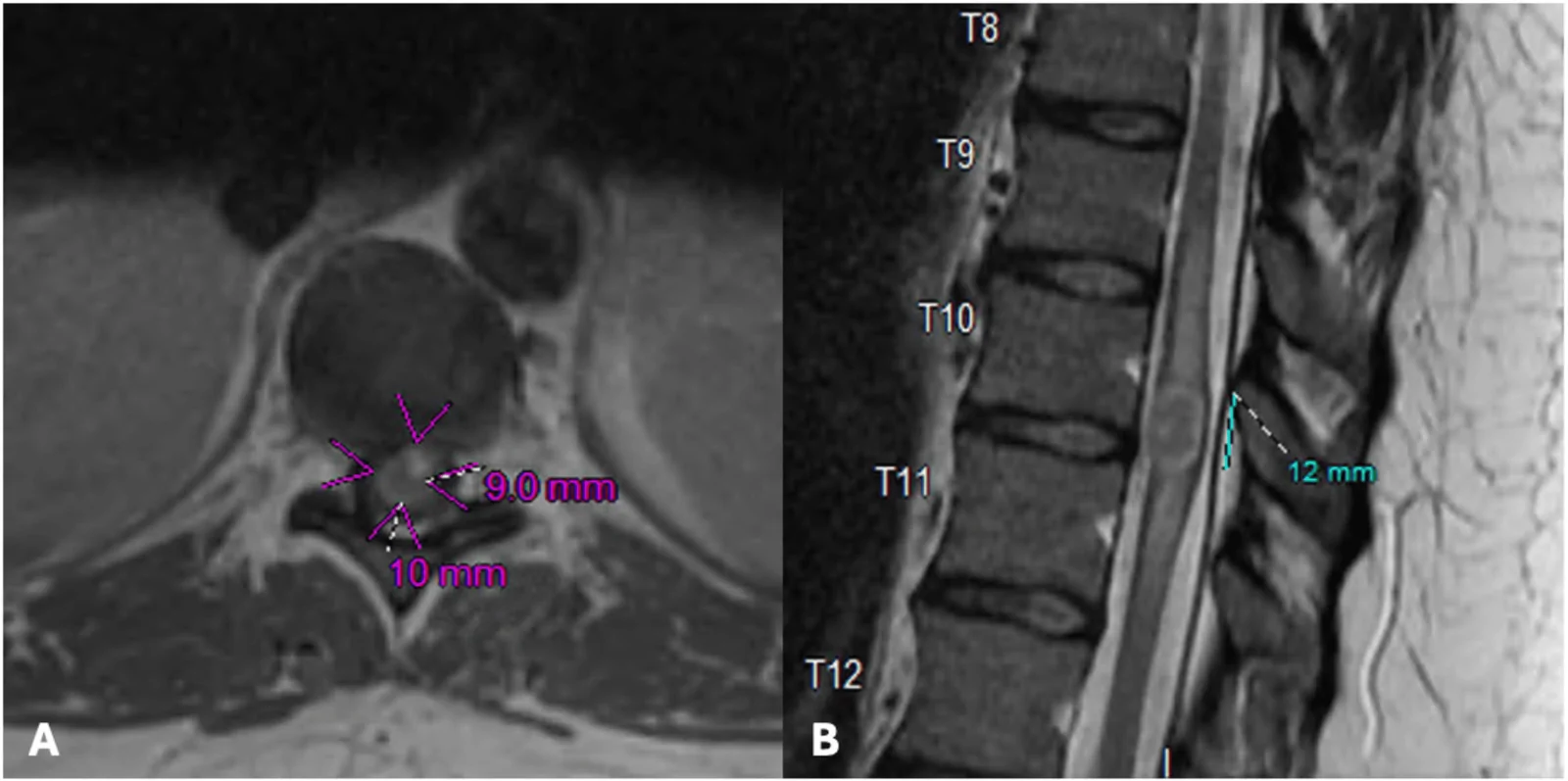
Pretreatment T1-weighted (A) axial and (B) sagittal MRI sequences of the spine demonstrating a 12 mm × 9 mm × 10 mm ISCM at the T10 to T11 vertebral level. ISCM, intramedullary spinal cord metastasis

MRI of the head demonstrated two intracranial masses, consistent with metastases, in the left corpus callosum and the left cavernous sinus measuring up to 17 mm × 16 mm × 12 mm and 20 mm × 19 mm × 22 mm, respectively (Figure 2).

**Figure 2:**
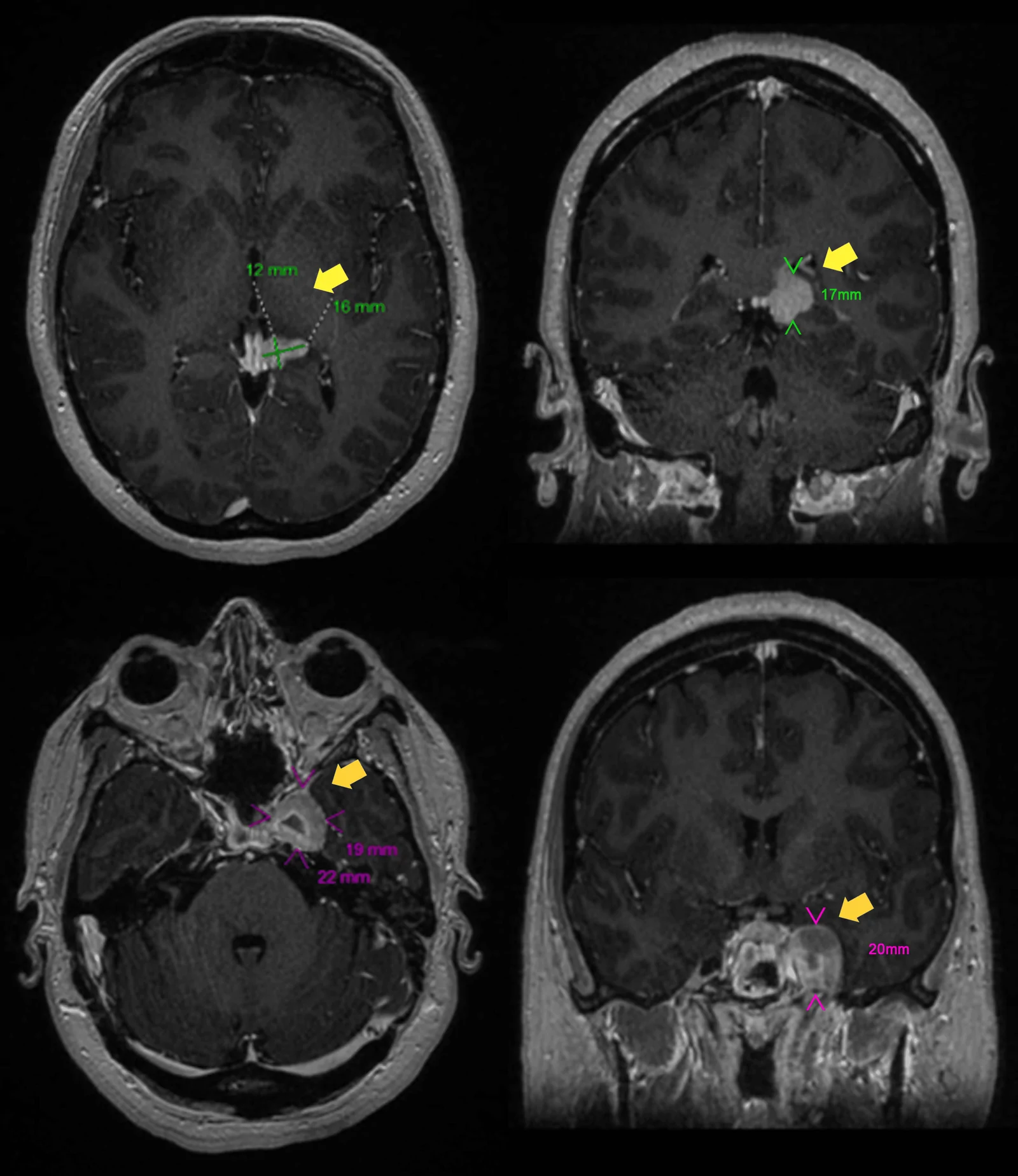
Pretreatment high-resolution T1-weighted cranial MRI sequences demonstrating a 17 mm × 16 mm × 12 mm left corpus callosum metastasis (yellow arrows) in (A) axial and (B) coronal views, and a 20 mm × 19 mm × 22 mm left cavernous sinus metastasis (orange arrows) in (C) axial and (D) coronal views.

On physical examination, she had decreased sensation in her right leg associated with decreased temperature sensation at presentation. Neurological examination demonstrated that her cranial nerves were otherwise full and intact except for left-sided facial numbness involving the V1, V2, and V3 distributions. Oral dexamethasone was initiated at a dose of 4 mg once daily to reduce peritumoral edema and alleviate neurologic symptoms. She underwent a stereotactic biopsy of the intracranial lesions, which revealed a spindle cell melanocytic neoplasm, B-Raf proto-oncogene, serine/threonine kinase (BRAF)-wild type. Stereotactic biopsy images are presented in Figure 3.

**Figure 3:**
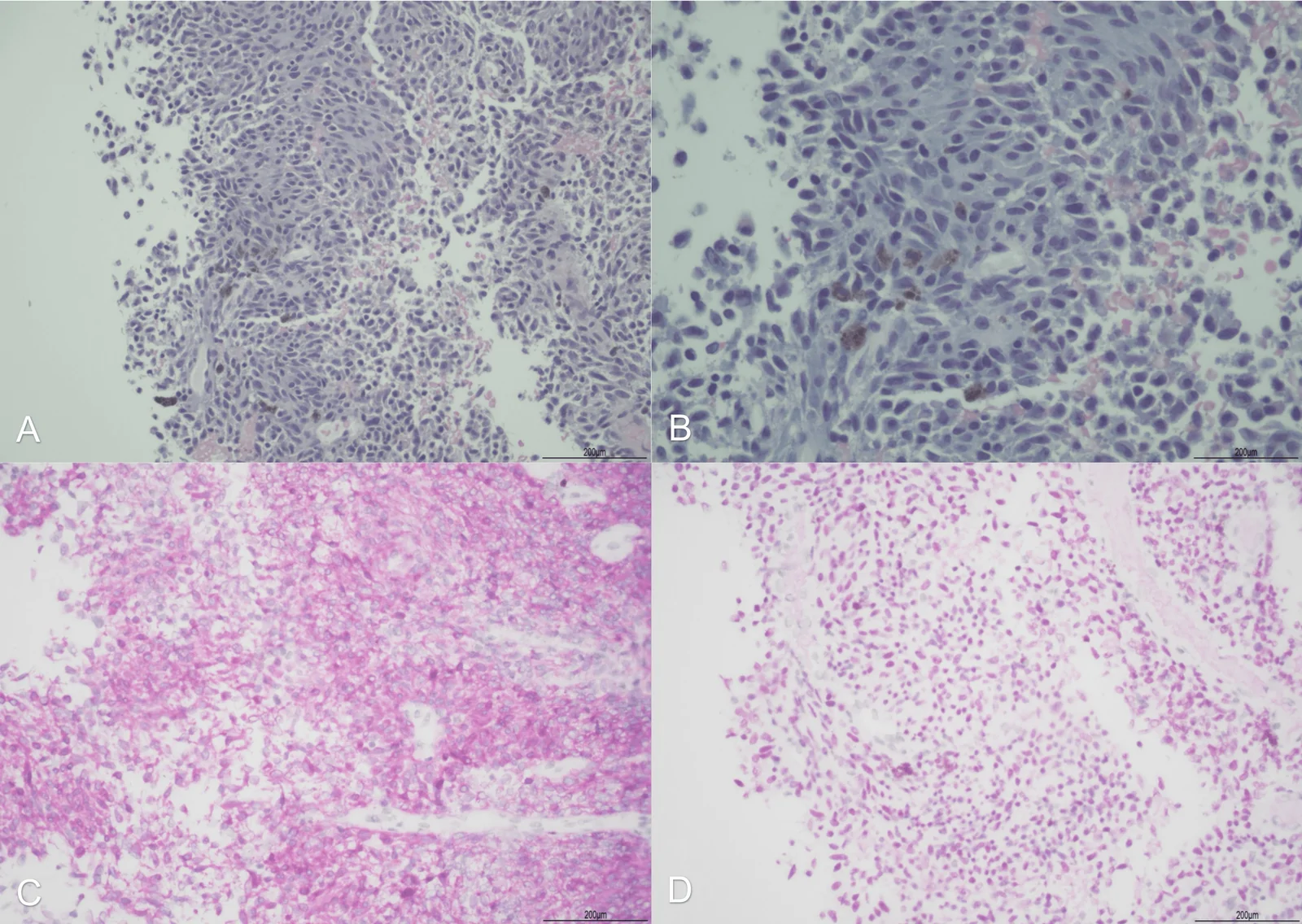
H&E stain at (A) low magnification (20× objective) and (B) high-power view (40× objective) demonstrating sheets of malignant melanocytes within a biopsy fragment with admixed subtle melanin pigment. (C) Immunohistochemical staining for Melan-A shows diffuse cytoplasmic positivity within the tumor cells, and (D) immunohistochemical staining for SOX-10 displays diffuse, strong nuclear positivity.

Lumbar puncture was performed, and cytology of the cerebrospinal fluid was nondiagnostic. There was no evidence of leptomeningeal disease on either cerebrospinal fluid cytology or spinal MRI. A PET-CT scan demonstrated no evidence of other distant metastases. An extensive total body skin examination, including the entire scalp, breast folds, vulvar area, anus, and webspaces of the hands and feet, was conducted by a dermatologist and demonstrated no suspicious malignant lesions. Ophthalmic evaluation by an ophthalmologist was negative for ocular nevi or melanoma.

Following multidisciplinary discussion, the patient was diagnosed with oligometastatic melanoma, and SBRT was recommended to the ISCM and stereotactic radiosurgery (SRS) to the intracranial metastases, as these were the only sites of gross disease. The patient was immobilized with the Body Pro-Lok ONE (CQ Medical, Avondale, PA, USA) system and simulated according to our institutional protocol for spine SBRT using 1.25 mm axial CT slices. The gross tumor volume (GTV) for the ISCM was delineated using the co-registered high-resolution T1- and T2-weighted axial MRI sequences. The volume of the GTV was 1.2 cc. No clinical target volume was used. A 2 mm margin was added to the GTV to create the planning target volume (PTV). A prescription dose of 30 Gy in 5 fractions, delivered over consecutive business days, was selected as it was felt to have the largest therapeutic window, balancing the probability of tumor control and normal tissue complication. A spinal cord dose constraint limiting the maximum dose to 0.035 cc to 30 Gy in 5 fractions per the American Association of Physicists in Medicine (AAPM) Task Group (TG) 101 report [8]. No PRV was used for the spinal cord to maximize the dose to the GTV since the tumor was within the spinal cord. Adding a PRV would have resulted in substantial tumor undercoverage, which may have increased the risk of local failure. The dosimetric objectives and achieved metrics for the target volumes and organs at risk are described in Table 1.

**Table 1:** Dosimetric values for the ISCM radiotherapy planning. Dmax, maximum dose to 0.035 cc; Dmin, minimum dose received by 99.5% of the volume; GTV, gross tumor volume; ISCM, intramedullary spinal cord metastasis; PTV, planning target volume; Vx Gy, volume receiving at least x Gy

Volumes	Clinical goals	Doses
GTV	V29.7 Gy >98%	80.7%
PTV	Dmin >50%	93.5%
	Dmax <150%	111.8%
	V27 Gy >95%	100.0%
	V30 Gy >99%	43.6%
Spinal cord	Dmax <30 Gy	29.97 Gy

Prioritizing the spinal cord dose constraint led to GTV undercoverage, with only 80.7% of the GTV receiving at least 29.70 Gy compared with the 98% planning goal; similarly, only 43.6% of the PTV received 30 Gy instead of the 99% coverage goal. The conformity index was 1.38, the gradient index was 15.31, and the homogeneity index was 1.15. The dose distribution from the SBRT plan for the ISCM is presented in Figure 4.

**Figure 4:**
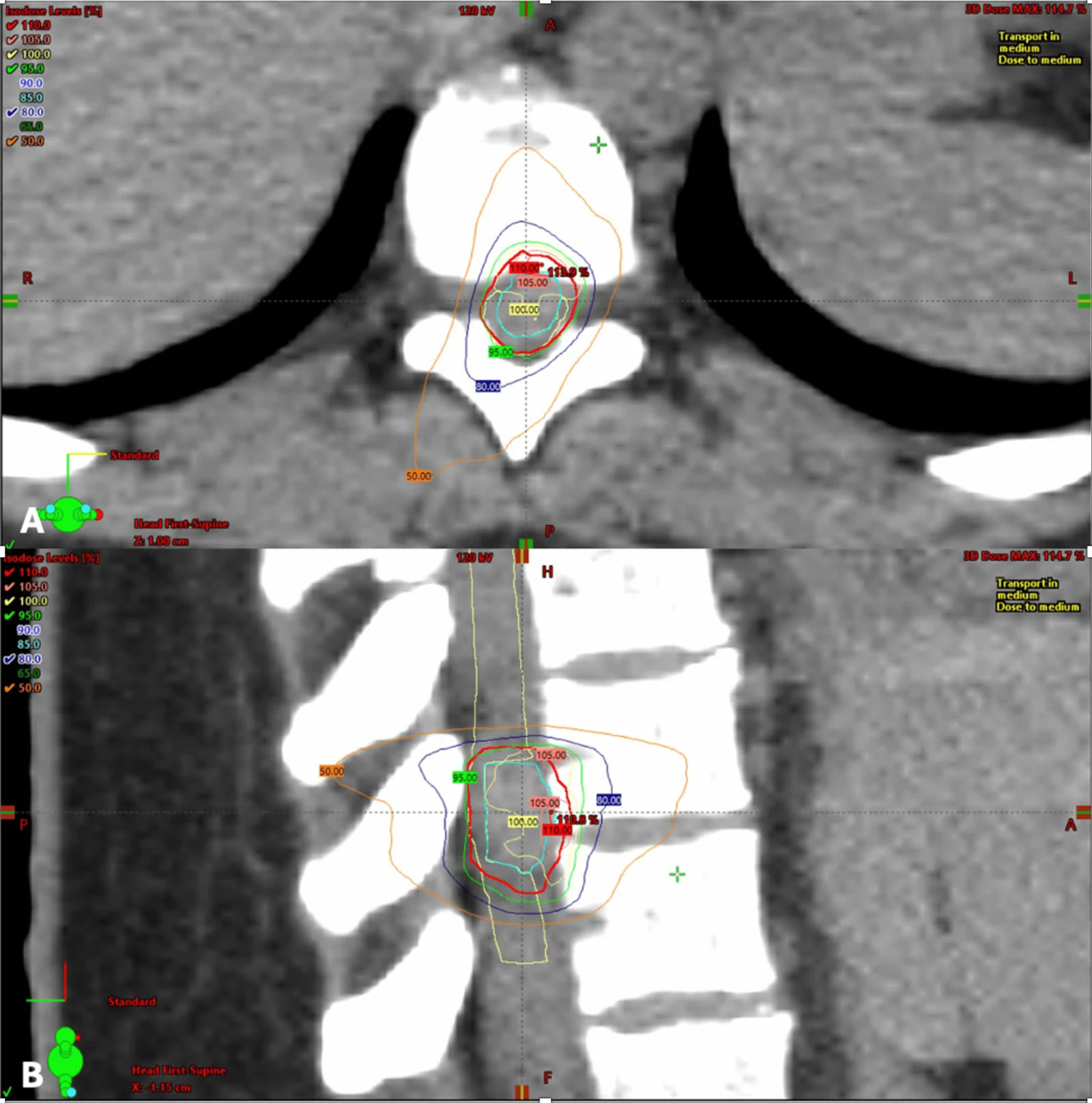
SBRT dose distribution showing the GTV (cyan) and the PTV (red) for the ISCM at the T10 to T11 vertebral level, as well as the spinal cord (yellow), in (A) axial and (B) sagittal views. GTV, gross tumor volume; ISCM, intramedullary spinal cord metastasis; PTV, planning target volume; SBRT, stereotactic body radiotherapy

The intracranial metastases were treated with SRS to a dose of 20 Gy in a single fraction. The patient was simulated in the supine position using an Encompass (QFix, Avondale, PA, USA) immobilization shell. A co-registered high-resolution T1-weighted contrast-enhanced MRI was used to delineate the GTVs, with a 1 mm margin added to generate the PTVs. Planning and delivery used a single-isocenter multitarget technique with HyperArc (Varian, a Siemens Healthineers company, Palo Alto, CA, USA) and Identify (Varian, a Siemens Healthineers company). The SBRT and SRS treatments were delivered on an Edge linear accelerator (Varian, a Siemens Healthineers company). Image guidance with cone-beam CT was performed before all fractions. Dexamethasone was gradually tapered after completion of radiotherapy.

At two weeks post-radiotherapy, the patient noted symptomatic improvement in her temperature sensation and facial numbness. At six weeks post-radiotherapy, a combination of ipilimumab and nivolumab was sequentially given for four cycles, followed by maintenance nivolumab. Dexamethasone was successfully discontinued prior to the initiation of immunotherapy. At eight weeks post-radiotherapy, she experienced worsening left-sided facial numbness and right leg hyperalgesia associated with some loss of sensation, which was attributed to a flare reaction or treatment response to immunotherapy. Her pain was relieved with oral hydromorphone 1 mg as needed. At 16 weeks post-radiotherapy, she also developed left lateral gaze vertical diplopia due to increased intracranial pressure. Dexamethasone was reinitiated, resulting in symptomatic improvement. Consequently, nivolumab was delayed until steroid discontinuation and then resumed. She subsequently developed immunotherapy-related hypothyroidism and adrenal insufficiency at five months and 10 months post-immunotherapy, respectively; both conditions were managed with hormone replacement therapy by an endocrinologist. At 25 months post-radiotherapy, the treated intracranial metastases and the ISCM at the T10 to T11 vertebral level decreased in size, with no progressive distant metastasis. Posttreatment MRI scans of the treated ISCM and intracranial metastases are shown in Figure 5 and Figure 6, respectively.

**Figure 5:**
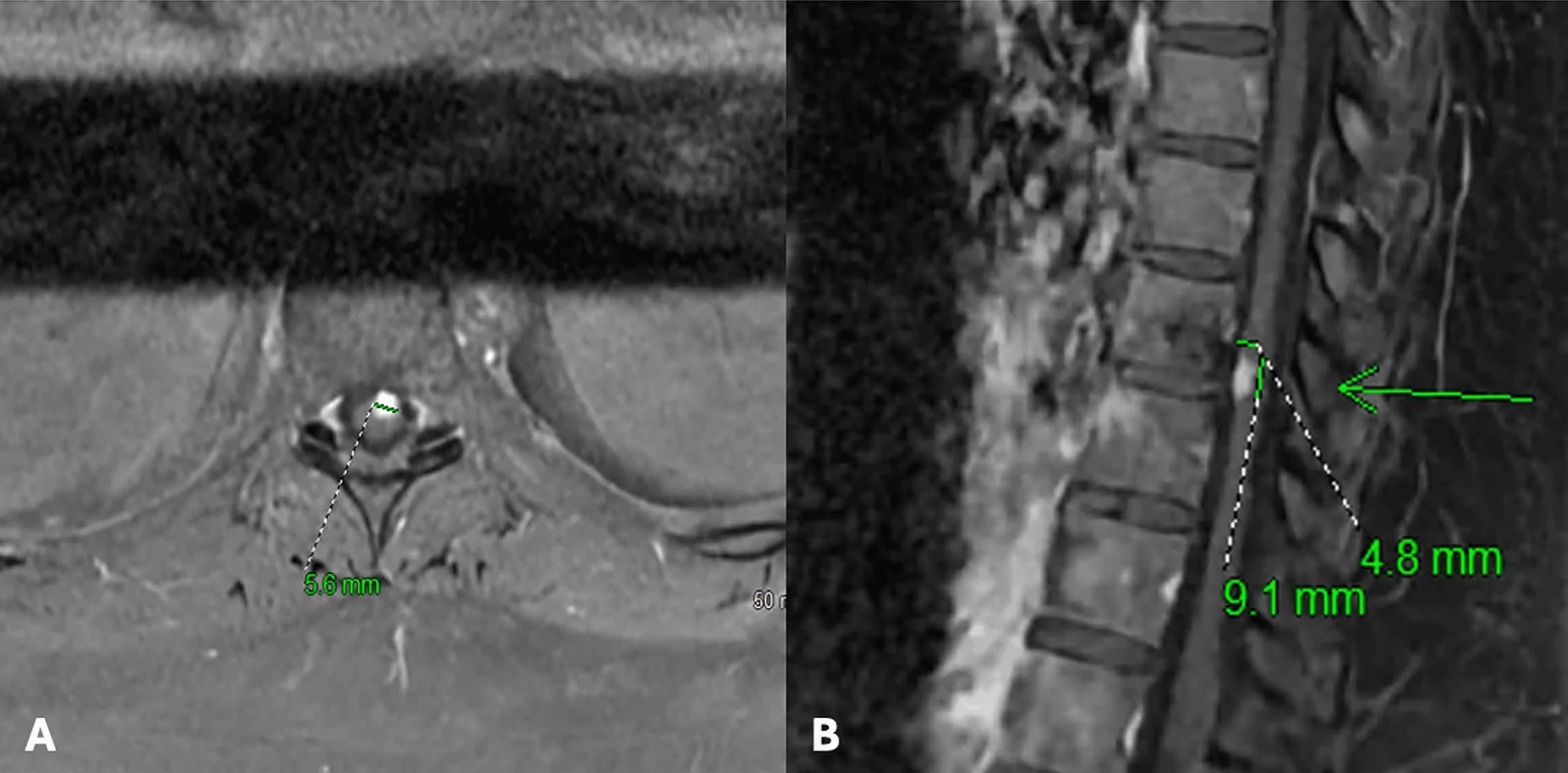
Posttreatment T1-weighted axial (A) and sagittal (B) MRI sequences of the spine demonstrating a 9.1 mm × 5.6 mm × 4.8 mm ISCM at the T10 to T11 vertebral level at 25 months post-SBRT. ISCM, intramedullary spinal cord metastasis; SBRT, stereotactic body radiotherapy

**Figure 6:**
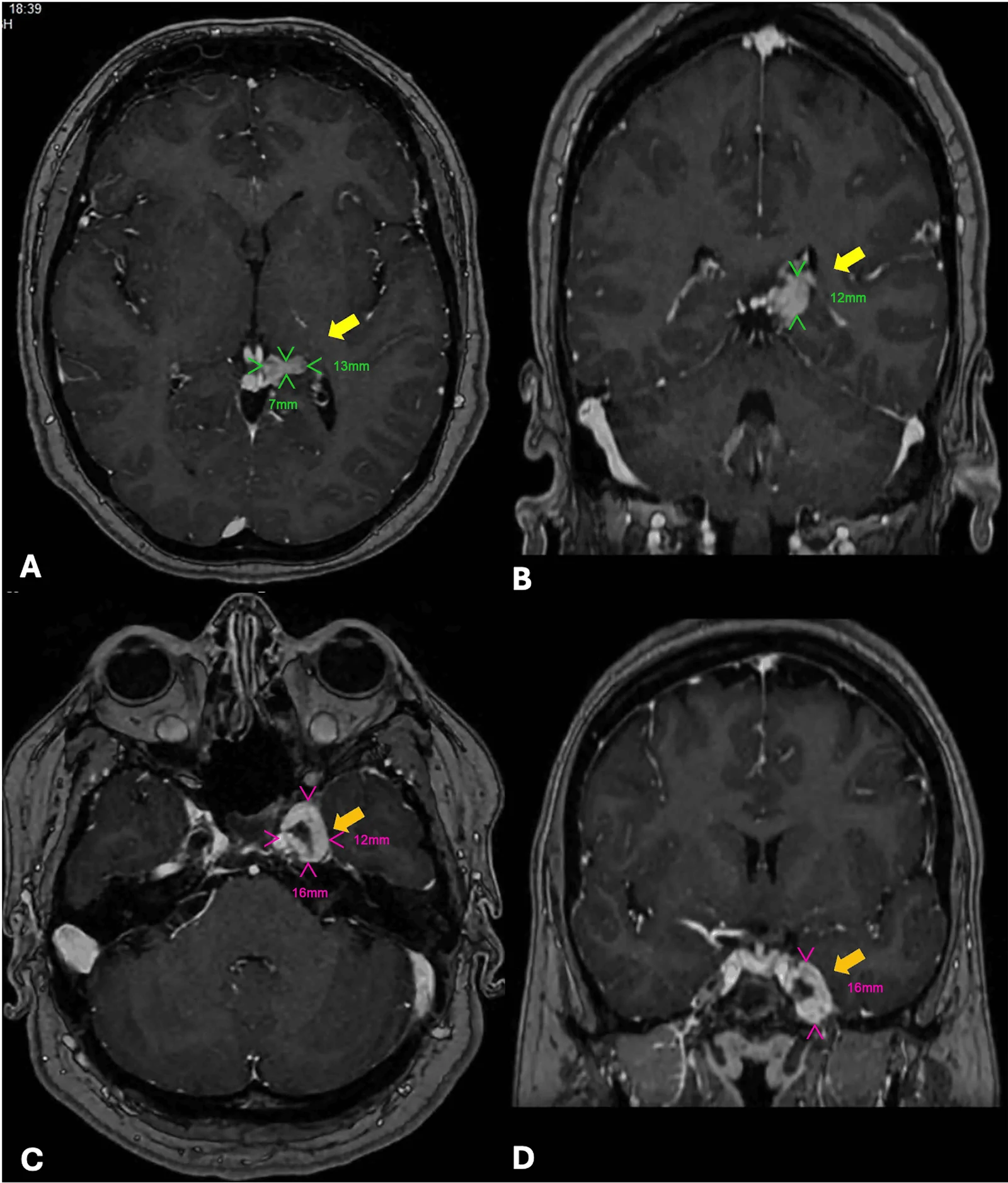
Posttreatment T1-weighted cranial MRI sequences demonstrating a 12 mm × 13 mm × 7 mm left corpus callosum metastasis (yellow arrows) in (A) axial and (B) coronal views, and a 16 mm × 12 mm × 16 mm left cavernous sinus metastasis (orange arrows) in (C) axial and (D) coronal views, at 25 months post-SRS. SRS, stereotactic radiosurgery

Posttreatment MRI of the spine and head demonstrated a decrease in the size of the tumors and improvement of peritumoral edema. As of her last follow-up at 25 months post-radiotherapy, she reported stable paresthesia and decreased temperature sensation involving her distal right leg and stable left-sided facial numbness with a Zubrod performance status of 1. No grade 3+ toxicity occurred secondary to SBRT or immunotherapy. The patient remained fully ambulatory throughout her treatment course.

## Discussion

This case report describes the treatment of a patient with oligometastatic melanoma of unknown primary with ISCM and synchronous intracranial metastases treated with SBRT and SRS, respectively. The standard of care for the management of ISCM is yet to be defined. The incidence of oligometastatic melanoma of unknown primary with ISCM and synchronous intracranial metastases at presentation is extremely rare. Several small retrospective studies and case series have demonstrated the safety and efficacy of SBRT in the management of ISCM [2,9,10]. In a retrospective study of 33 patients (46 ISCMs) treated primarily with robotic radiosurgery (median dose of 16 Gy delivered to the 70% isodose line; median tumor volume = 0.7 cc), Ehret et al. reported a 79% local control rate with improved or stable neurological symptoms in 57% of cases at a median follow-up of 8.5 months [9]. Similarly, a case series of five patients with ISCM from the Oscar Lambret Centre treated with SBRT (median dose of 30 Gy delivered to the 73% to 85% isodose line; mean tumor volume = 1.4 cc; no spinal cord PRV) demonstrated 50% complete and 25% partial response rates at a median follow-up of 23 months [10]. Neither study reported radiation myelitis (RM) or any significant treatment-related toxicity [9,10]. This case demonstrates durable local control without significant SBRT-related toxicity at 25 months follow-up, consistent with prior studies. However, comparing clinical outcomes in oligometastatic melanoma with ISCM remains difficult due to the rarity of the disease and the overall histological heterogeneity in published series [2,9,10]. For instance, melanoma only accounted for 8.3% of ISCM cases in a systematic review by Carvajal et al., and the details regarding systemic therapy were limited [2]. The role of SBRT continues to evolve in patients with oligometastatic melanoma with ISCM in parallel with advances in systemic therapy, particularly immunotherapy. As immunotherapy improves survival for patients with metastatic melanoma [7], the clinical priority shifts from low-dose palliative radiotherapy to high-precision, higher-dose radiotherapy to achieve better long-term, durable control while minimizing potential side effects [11]. However, variations in SBRT dose fractionation exist, and there is no consensus yet regarding the preferred dose for ISCM. Small retrospective studies and case reports have reported differing regimens ranging from 1 to 13 fractions, with a median dose of 17 Gy based on a systematic review and meta-analysis by Carvajal et al. [2]. The recommended spine SBRT doses by the European Society for Radiotherapy and Oncology and National Cancer Network for single-fraction and 3-fraction regimens are 16-24 Gy and 24-30 Gy, respectively [11,12]. Although these regimens provide a higher biologically effective dose, they exceed recommended spinal dose constraints per AAPM TG 101 (14.0 Gy and 21.9 Gy for the single- and 3-fraction regimens, respectively). Therefore, adhering to these constraints can be challenging when the tumor is within the spinal cord, as in the case of ISCM. Ultimately, optimizing the therapeutic window by balancing the probabilities of tumor control and normal tissue complication is an important concept in selecting an appropriate dose fractionation. In the present case, a dose of 30 Gy in 5 fractions was prescribed while prioritizing the AAPM TG 101 5-fraction maximum dose limit of 30 Gy to the spinal cord [8]. This fractionation was selected because it was felt to maximize the therapeutic window to deliver an adequate dose to the tumor while sparing the spinal cord.

There is growing evidence supporting the safe delivery of SBRT for ISCM when adhering to consensus spinal dose constraints [2,8-10]. A spinal cord PRV is commonly used in spine SBRT due to concerns regarding spinal cord motion [13,14]. The spinal cord PRV accounts for the physiologic motion of the spinal cord within the cerebrospinal fluid during the cardiac or respiratory cycle [13]. This is crucial in spine SBRT due to the proximity of the PTV to the spinal cord, where small changes in positioning can lead to significant dosimetric differences due to the steep dose gradients characteristic of SBRT. Therefore, in clinical scenarios where the tumor is closely related to the spinal cord and accounting for spinal cord motion is required, a fractionated regimen may be preferred [13]. Different models used varying anatomical definitions of the spinal cord or surrogates to predict the risk of RM [14,15]. Sahgal et al. used the thecal sac as a surrogate for the spinal cord [14], whereas Jackson et al. did not use a PRV for the spinal cord when it was delineated using a myelogram prior to CT simulation and added a 1 mm PRV when using a co-registered MRI [15]. Additionally, Lucido et al. used the true spinal cord as the OAR without a PRV and adhered to AAPM TG 101 dose limits for 1, 3, and 5 fractions, which also yielded a low incidence of RM [16]. These findings suggest that SBRT may be safe when adhering to recommended spinal dose constraints regardless of whether a PRV is applied [14-16]. The omission of a spinal cord PRV may be reasonable, particularly for patients with ISCM, where inadequate tumor coverage leading to local failure could be devastating. This approach should be undertaken with caution and implemented on a case-by-case basis, weighing the goals of durable local control and side effects. In this case, despite omitting the spinal cord PRV, substantial undercoverage of the GTV and PTV occurred because spinal cord dose constraints were prioritized over target coverage to ensure patient safety. Nonetheless, the patient in the present case continued to show no evidence of disease progression in the treated sites and no reported SBRT-related toxicity at 25-month follow-up.

A systematic review by Cattaneo et al. demonstrated that combining SBRT with immunotherapy does not increase the risk of SBRT-related toxicity compared with immunotherapy alone [17]. Furthermore, concurrent and sequential use of SBRT with immunotherapy showed comparable rates of grade 3+ toxicity; pneumonitis was the most common adverse event during concurrent therapy, whereas colitis, rash, and fatigue predominated with sequential administration [17]. Although temporal sequencing of SBRT and immunotherapy appears to influence immune responses, the interpretation of published outcomes remains challenging due to study heterogeneity and variable reporting of SBRT parameters, toxicity grading, and treatment schedule [17]. Various mechanisms have been proposed regarding the synergistic effect of SBRT combined with immunotherapy. These include promoting infiltration of resident and circulating T cells, as well as suppression of regulatory T cells and myeloid-derived suppressor cells within the tumor microenvironment to enhance systemic immune activation, particularly with PD-1 blockade [18]. Additionally, radiation-induced upregulation of PD-L1 on tumor and stromal cells enhances sensitivity to immune checkpoint inhibitors [19]. In this case, since SBRT and immunotherapy were delivered in close succession, we cannot exclude the possibility of synergy between SBRT and immunotherapy that may have contributed to the favorable overall treatment response.

## Conclusions

SBRT for ISCM in oligometastatic melanoma of unknown primary is feasible and provided durable local control while preserving neurological function after 25 months of follow-up. There was no significant SBRT-related toxicity, although the patient developed immunotherapy-related side effects. Omission of a spinal cord PRV in patients with ISCM may maximize the dose to the GTV to improve local control without increasing the risk of RM. This approach should be individualized and weigh the goals of durable local control and risk of toxicity. Further research is needed to establish the optimal dose fractionation, PRV strategy, and timing of immunotherapy for ISCM. Larger prospective studies are warranted.
